# Ruxolitinib for steroid-refractory chronic graft-versus-host disease: Japanese subgroup analysis of REACH3 study

**DOI:** 10.1007/s12185-024-03850-9

**Published:** 2024-10-03

**Authors:** Souichi Shiratori, Kentaro Fukushima, Yasushi Onishi, Noriko Doki, Tatsunori Goto, Masaya Okada, Hirohisa Nakamae, Yoshinobu Maeda, Koji Kato, Takayuki Ishikawa, Tadakazu Kondo, Masako Toyosaki, Takashi Ikeda, Naoyuki Uchida, Akio Maki, Fumika Shimada, Takeshi Tajima, Tommaso Stefanelli, Takanori Teshima

**Affiliations:** 1 Department of Hematology, Faculty of Medicine, Hokkaido University, Sapporo, Japan; 2 Department of Hematology and Oncology, Osaka University Graduate School of Medicine, Osaka, Japan; 3 Department of Hematology, Tohoku University Hospital, Miyagi, Japan; 4 Tokyo Metropolitan Cancer and Infectious Diseases Center Komagome Hospital, Tokyo, Japan; 5 Japanese Red Cross Aichi Medical Center Nagoya Daiichi Hospital, Aichi, Japan; 6 Hyogo College of Medicine, Kansai Medical University Medical Center, Hyogo, Japan; 7 Department of Hematology, Osaka Metropolitan University, Osaka, Japan; 8 Department of Hematology, Oncology and Respiratory Medicine, Graduate School of Medicine, Dentistry, and Pharmaceutical Sciences, Okayama University, Okayama, Japan; 9 Department of Hematology, Oncology and Cardiovascular Medicine, Kyushu University Hospital, Fukuoka, Japan; 10 Department of Hematology, Kobe City Medical Center General Hospital, Hyogo, Japan; 11 Department of Hematology and Oncology, Graduate School of Medicine, Kyoto University, Kyoto, Japan; 12 Department of Hematology and Oncology, Tokai University School of Medicine, Kanagawa, Japan; 13 Division of Hematology and Stem Cell Transplantation, Shizuoka Cancer Center, Shizuoka, Japan; 14 Department of Hematology, Toranomon Hospital, Tokyo, Japan; 15 Novartis Pharma K.K, Tokyo, Japan; 16 Novartis Pharma AG, Basel, Switzerland

**Keywords:** Chronic graft-versus-host disease, Ruxolitinib, REACH3, JAK inhibitor, Japanese

## Abstract

Ruxolitinib, a Janus kinase (JAK1–JAK2) inhibitor, has demonstrated safety and efficacy in patients with graft-versus-host disease (GvHD). This phase 3 randomized trial (REACH3) evaluated the efficacy and the safety of ruxolitinib 10 mg twice daily compared with investigator-selected best available therapy (BAT) in a subgroup of Japanese patients (*n* = 37) with steroid-refractory or dependent (SR/D) chronic GvHD. At data cut-off, treatment was ongoing in 17 patients and discontinued in 20. The overall response rate (complete or partial) at week 24 was greater with ruxolitinib than BAT (50% vs. 20%; odds ratio, 4.13 [95% CI, 0.90–18.9]). The best overall response rate (complete or partial response at any time point up to week 24) was higher with ruxolitinib than BAT (68.2% vs. 46.7%; odds ratio, 2.69 [95% CI, 0.66–10.9]). Ruxolitinib led to longer median failure-free survival than BAT (18.6 months vs. 3.7 months; hazard ratio, 0.34; [95% CI, 0.14–0.85]). The most common grade ≥ 3 adverse events up to week 24 were anemia (ruxolitinib: 22.7%; BAT: 6.7%) and pneumonia (22.7% and 20.0%, respectively). Ruxolitinib showed a higher response rate and improvement in failure-free survival in Japanese patients with SR/D chronic GvHD, with a safety profile consistent with the overall study population.

**Supplementary Information:**

The online version contains supplementary material available at 10.1007/s12185-024-03850-9.

## Introduction

Hematopoietic stem cell transplantation (HSCT) remains the only curative therapeutic approach for medical conditions that result in bone marrow failure [1]. However, significant treatment-related mortality associated with HSCT is one of the major limitations restricting its wider adoption [2]. Chronic graft-versus-host disease (cGvHD) is a frequent medical complication of allogeneic HSCT that manifests as an autoimmune-like inflammatory disease which can have a devastating impact on overall health and quality of life (QoL) [3]. cGvHD affects 50% of long-term marrow transplant survivors and is fatal in 20–40% of the affected patients [4].

At Japanese centers, the 2-year cumulative incidence of cGvHD was found to be 35.4% (*n* = 145/406) in a prospective, longitudinal study conducted in HSCT patients with a median onset of 4.7 months after transplant [5]. Interestingly, Japanese patients had a lower incidence of cGvHD (bone marrow transplantation, 15% vs 30% [*p* = 0.002]; peripheral blood stem cell transplantation, 37% vs 45% [*p* < 0.001]) and experienced more frequent cGvHD of the mouth, eyes, and liver and less-frequent gastrointestinal cGvHD when compared with White patients [6].

Steroid therapy, such as systemic glucocorticoids, remains as the standard first-line treatment for cGvHD [7, 8]. However, only 40–50% of patients respond adequately to this treatment, and over half of these patients become steroid-resistant or -dependent (SR/D), requiring second-line treatment [9]. High doses and prolonged use of steroids may lead to significant side effects, such as opportunistic infections, diabetes, myopathy, and osteonecrosis, thereby affecting the patient’s QoL [10]. Secondary treatment options for cGvHD across the world include extracorporeal photopheresis (ECP), ibrutinib, rituximab, pentostatin, mycophenolate mofetil (MMF), and mammalian target of rapamycin (mTOR) inhibitors. In Japan, ECP, ibrutinib and MMF are the only approved second-line therapies for treatment of cGvHD. Both drugs were approved based on single group studies [11–13] and there were no successful randomized study data available. Therefore, no standard second-line treatment has been established.

Janus kinases (JAKs) are intracellular tyrosine kinases that play a significant role in acute and cGvHD pathogenesis via variety of cytokines signaling [14, 15]. Ruxolitinib is an orally administered selective JAK1/2 inhibitor approved for treatment of patients with SR-acute graft-versus-host disease (aGvHD) and/or SR-cGvHD in several countries, including Japan. In a retrospective study, ruxolitinib demonstrated high overall response rate (ORR) in steroid-resistant patients with GvHD highlighting the potential clinical value of this drug for salvage therapy in this targeted population [16]. In further prospective studies, ruxolitinib has demonstrated significant clinical improvements among patients with grade II to IV SR-acute GvHD in phase 2 REACH1 study (NCT02953678) and phase 3 REACH2 study (NCT02913261) [17, 18].

REACH3, a phase 3 study, evaluated efficacy and safety of ruxolitinib 10 mg BID vs best available therapy (BAT) in patients with moderate or severe SR/D cGvHD. This study met the primary endpoint with ruxolitinib demonstrating significantly higher ORR at week 24 (49.7% vs 25.6%; odds ratio (OR), 2.99; *p* < 0.001), longer median failure-free survival (> 18.6 months vs 5.7 months; hazard ratio (HR), 0.37; *p* < 0.001), and higher symptom response (24.2% vs 11.0%; OR, 2.62; *p* = 0.001) when compared with BAT in patients with SR-cGvHD [19]. Here, in this subgroup analysis, therapeutic efficacy and clinical outcomes associated with ruxolitinib in patients with cGvHD among the Japanese population are presented.

## Methods

### Study design

The study design and patient eligibility criteria of REACH3 study have been published previously [19]. Briefly, REACH3 (ClinicalTrials.gov number: NCT03112603) was an international, multicenter, open-label, randomized phase 3 study which evaluated the efficacy and safety of ruxolitinib 10 mg twice daily (BID) vs BAT in 329 patients with moderate or severe SR/D cGvHD. After randomization (1:1), eligible patients received either ruxolitinib 10 mg BID or investigator chosen BAT for at least 6 cycles (28 days per cycle) and were stratified by cGvHD severity as defined by National Institutes of Health (NIH) consensus staging criteria [20] and determined to be SR/D cGvHD per NIH consensus criteria [21]. Corticosteroids with or without calcineurin inhibitors (CNIs) were allowed to continue and infection prophylaxis was allowed as per the local institutional guidelines. Steroid tapering was allowed after patients had a complete or partial response (CR or PR). Tapering of CNI/ruxolitinib was allowed after week 24 and after patients achieved CR or PR. BAT options included ECP, low-dose methotrexate (MTX), MMF, mTOR inhibitors (everolimus or sirolimus), infliximab, rituximab, imatinib, pentostatin, or ibrutinib. Initiation of new BAT was allowed before week 24 due to treatment failure indicated by lack of response, unacceptable side effects, or cGvHD flare. Crossover to ruxolitinib from BAT was allowed after week 24 in patients who had disease progression, mixed or unchanged response, or unacceptable side effects. These patients were allowed to continue corticosteroids with or without CNIs as per standard of care, with required cessation before switching to ruxolitinib treatment.

The data cut-off for the primary analysis was May 08, 2020. Primary efficacy period was from start of randomized treatment to week 24 followed by extension period up to week 156. After week 24, patient study visits were scheduled every 12 weeks up to week 156 or end of treatment, whichever occurred first. Patients who discontinued the study treatment before week 156 entered long-term survival follow-up period and visits were scheduled every 3 months until week 156 and reporting of new cGvHD therapy until week 156 were completed. At 30 days after the last dose of study treatment, a safety follow-up visit was scheduled to collect data on survival, progression, and safety outcomes. Patients who crossed over to ruxolitinib were followed up until completion of treatment. Randomization (1:1) was implemented for the global population and not stratified by geography. Therefore, the patient number for Japanese subgroup may not exactly reflect the 1:1 allocation.

The study was designed and conducted in accordance with the guidelines for Good Clinical Practice of the International Council for Harmonization, with applicable local regulations, and with the principles of the Declaration of Helsinki. The protocol was approved by the relevant institutional review board, independent ethics committee, or research ethics board at each participating center. Signed informed consent was obtained from the participating patient, parent, or guardian.

### Patients

The patient population was ≥ 12 years old, received allogeneic HSCT, had evidence of myeloid and platelet engraftment (absolute neutrophil count > 1000/mm^3^ and platelet count > 25,000/mm^3^) and displayed moderate or severe SR/D cGvHD based on NIH consensus criteria [20, 21]. Patients with a relapse of primary cancer or graft loss within 6 months before treatment initiation, or an active, uncontrolled infection were excluded. Patients who received 2 or more systemic therapies for cGvHD in addition to corticosteroids with or without CNIs were also excluded.

### Endpoints

The endpoints of REACH3 study have been described in detail previously [19]. In brief, the primary endpoint was ORR (defined as a complete or partial response according to 2014 NIH consensus response criteria) [22] at week 24 and the key secondary endpoints were failure-free survival (FFS, defined as time from the date of randomization to the earliest of recurrence of underlying disease, start of new systemic treatment for cGvHD, or death) and the response in the modified Lee Symptom Scale (mLSS) score [23, 24] (defined as a ≥ 7-point reduction from baseline in total symptom score (TSS), which measures the symptoms of cGvHD on a scale of 0 to 100, with higher scores indicating worse symptoms) at week 24. In this study, the LSS was modified (i.e., mLSS) so that patients reported on symptom severity rather than “bother” and had a recall period of 1 week instead of 1 month. Other secondary endpoints included best overall response (BOR), duration of response (DOR), change in corticosteroid dose over time, overall survival, and changes in QoL measures (Functional Assessment of Cancer Therapy–Bone Marrow Transplant: FACT-BMT v4.0 and EuroQoL 5-dimension 5-level: EQ-5D-5L).

Safety analyses included patients who received at least one dose of treatment; safety data up to week 24 are presented to ensure similar exposure between arms.

Pharmacokinetics (PK) were assessed by collecting plasma samples. Serial blood samplings were performed at predose and 0.5, 1.0, 1.5, 4, 6, and 9 h postdose on day 1 and day 15 for a subset of patients to calculate PK parameters in plasma with a non-compartmental method using Phoenix WinNonlin® (Pharsight, Mountain View, CA), maximum plasma concentration (C_max_), time to reach the maximum concentration (T_max_), and area under the plasma concentration–time curve to the last measurable concentration (AUC_last_). Sparse samplings including measurement of trough concentrations were conducted in all patients during the study. Plasma concentrations were measured using a validated liquid chromatography–tandem mass spectrometry method with the lower limit of quantification at 0.500 ng/mL.

### Statistical analysis

In the REACH3 study, a target of 324 patients expected to achieve 90% power for the ORR and FFS; it was planned to include at least 30 patients from Japan. Efficacy analysis was performed for the full analysis set composed of all patients who underwent randomization, and safety analysis for the safety analysis set including all patients who received at least 1 dose of study treatment. The PK analysis set (PAS) included all patients who provided at least one evaluable PK concentration. The PAS was used for all PK data analysis. Results for the subgroup of Japanese patients enrolled to the global REACH3 study were analyzed descriptively. No formal statistical test of hypotheses was planned and performed for the subgroup, and the point estimates were provided with 95% confidence interval (CI).

## Results

### Patient disposition and baseline characteristics

**Fig. 1:**
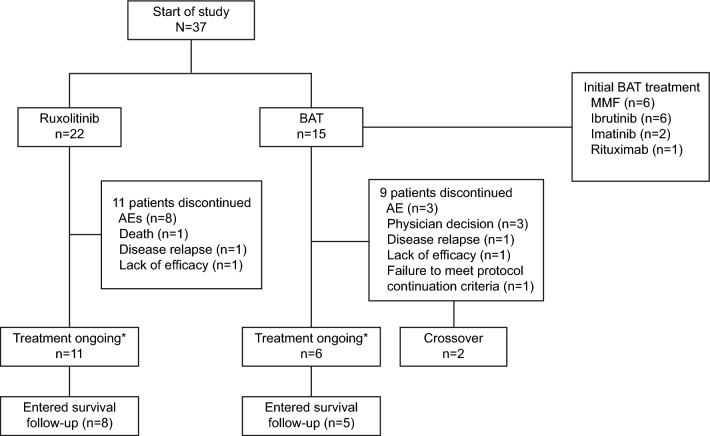
Patient disposition. *Ongoing treatment and/or assessments during the main treatment period at the data cut-off date May 08, 2020. *AE* adverse events, *BAT* best available therapy, *MMF* mycophenolate mofetil

In this subgroup analysis of REACH3 study, 37 Japanese patients were randomized to receive either ruxolitinib (*n* = 22) or BAT (*n* = 15) (Fig. 1). At data cut-off (May 08, 2020), study treatment was ongoing in 11 (50%) patients in ruxolitinib group vs 6 (40%) patients in BAT group; 11 (50%) and 9 (60%) patients in the ruxolitinib and BAT groups discontinued the randomized treatment. Reasons for treatment discontinuation (ruxolitinib vs BAT) included adverse events (36.4% vs 20%), lack of efficacy (4.5% vs 6.7%), disease relapse (4.5% vs 6.7%), and death (4.5% vs 0%). Of the 15 patients in the BAT group, 2 (13.3%) participants crossed over to ruxolitinib on or after week 24.

Demographics and baseline characteristics of the patients were similar among both the treatment groups (Table 1). Among the Japanese patients who participated in REACH3 study, median age was 48.5 years (range, 15–68) and 40 years (range, 16 − 66) in ruxolitinib and BAT groups, respectively. Patients who had severe cGvHD at baseline were 75.7% that was numerically higher than the overall population in REACH3 study (56.8%). The stem cell sources were bone marrow (45.5%, 66.7%), peripheral blood (50.0%, 20.0%), and single cord blood (4.5%, 13.3%) in the ruxolitinib vs BAT groups, respectively. The proportions of HSCT from HLA-matched donors were 52.2% in the ruxolitinib group and 37.5% in the BAT group. Median time from the onset of cGvHD to randomization was 28.9 weeks (5.9–93.0) in ruxolitinib and 24 weeks (2.6–46.0) in BAT groups. The most common reason for diagnosis of SR/D cGvHD was lack of response or disease progression after prednisone treatment in 36.4% of patients in the ruxolitinib group and 13.3% of patients in the BAT group and overall, 37.8% of patients had steroid dependency. Prior medications and organ baseline scores are presented in Supplementary Table 1. The most common organ involvement in Japanese patients was lung (68.2% vs 66.7%) that was numerically higher than the overall population (44.8% vs 40.9%) in ruxolitinib versus BAT groups. In the overall population, skin was the most common organ involved (73.3% vs 68.9%) while this was lower in Japanese population (50.0% vs 60.0%).

**Table 1:** Demographics and clinical characteristics of the patients at baseline (Full analysis set)

Characteristic	Ruxolitinib (*n* = 22)	BAT (*n* = 15)	Total (*n* = 37)
Age, median (range), years	48.5 (15.0 − 68.0)	40.0 (16.0 − 66.0)	48.0 (15.0 − 68.0)
12 to < 18 years, *n* (%)	1 (4.5)	2 (13.3)	3 (8.1)
18 to 65 years, *n* (%)	18 (81.8)	12 (80.0)	30 (81.1)
> 65 years,* n* (%)	3 (13.6)	1 (6.7)	4 (10.8)
Sex			
Male,* n* (%)	15 (68.2)	9 (60.0)	24 (64.9)
SR-cGvHD severity,* n* (%)^a^			
Moderate	5 (22.7)	4 (26.7)	9 (24.3)
Severe	17 (77.3)	11 (73.3)	28 (75.7)
Refractory/dependent criteria,* n* (%)			
Lack of response or disease progression after prednisone ≥ 1 mg/kg/day for 1 week	8 (36.4)	2 (13.3)	10 (27.0)
Disease persistence without improvement despite continued treatment with prednisone	6 (27.3)	7 (46.7)	13 (35.1)
Increase to prednisone > 0.25 mg/kg/day after 2 unsuccessful attempts to taper: steroid dependency	8 (36.4)	6 (40.0)	14 (37.8)
Stem cell source,* n* (%)			
Bone marrow	10 (45.5)	10 (66.7)	20 (54.1)
Peripheral blood	11 (50.0)	3 (20.0)	14 (37.8)
Single cord blood	1 (4.5)	2 (13.3)	3 (8.1)
Donor type^b^,* n* (%)			
Related	12 (52.2)	6 (37.5)	18 (46.2)
Unrelated	11 (47.8)	10 (62.5)	21 (53.8)
Donor CMV status^b^,* n* (%)			
Negative	5 (21.7)	6 (37.5)	11 (28.2)
Positive	17 (73.9)	9 (56.3)	26 (66.7)
Missing	1 (4.3)	1 (6.3)	2 (5.1)
Recipient CMV status,* n* (%)			
Negative	7 (31.8)	6 (40.0)	13 (35.1)
Positive	15 (68.2)	9 (60.0)	24 (64.9)

^a^Severity was graded according to NIH consensus staging criteria at screening

^b^Some patients received more than one transplant

*BAT* best available therapy, *cGvHD* chronic graft-versus-host disease, *CMV* cytomegalovirus

The most common initial BAT selected in the study is presented in Supplementary Table 2. The underlying diseases, malignant in majority of patients, primarily included acute myeloid leukemia (63.6%, 40%) and myelodysplastic syndrome (22.7%, 20.0%) in the ruxolitinib vs BAT groups, respectively (Supplementary Table 3).

### Pharmacokinetics

PK parameters (C_max_, T_max_, and AUC_last_) were obtained in 1 adolescent and 2 adult Japanese patients in the ruxolitinib group on each sampling day of day 1 and day 15. The ranges of individual values of C_max_, T_max_, and AUC_last_ were 152–312 ng/mL, 0.483–0.667 h, and 595–1400 ng∙h/mL on day 1, and 281–451 ng/mL, 0.483–2.00 h, and 849–2190 ng∙h/mL on day 15, respectively. Trough concentrations in Japanese patients in the ruxolitinib group were obtained postdose up to week 24. The individual data ranged from 10.9 to 196 ng/mL (*n* = 2 to 17). The Japanese PK data were comparable to the individual range observed in non-Japanese patients.

### Efficacy

**Fig. 2:**
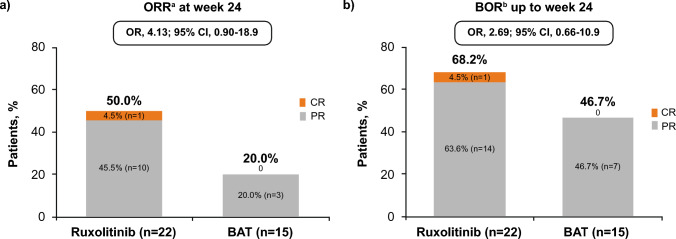
**a** Overall response rate (ORR) and **b** best overall response (BOR) up to week 24. **a** Overall response rate at week 24. ORR is defined as proportion of patients who achieved CR or PR according to 2014 NIH consensus criteria. **b** Best overall response rate up to week 24. BOR is defined as proportion of patients who achieved CR or PR at any time point up to week 24 or the start of additional systemic therapy for cGvHD. *BAT* best available therapy, *BOR* best overall response, *CR* complete response, *n* number of patients who are at the corresponding category, *OR* odds ratio, *ORR* overall response rate, *PR* partial response

The primary endpoint of ORR at week 24 was numerically higher with ruxolitinib than with BAT (50.0% [11/22] vs 20.0% [3/15]; OR, 4.13; 95% CI, 0.90–18.9; Fig. 2a) and ORR at week 12 was 45.5% [10/22] in ruxolitinib group relative to 33.3% (5/15 in BAT group [OR, 1.78; 95% CI, 0.45–7.15]). The number of responders at week 24 in the BAT group was 2 patients for MMF and 1 each for imatinib. The BOR, up to week 24, was observed in 68.2% (15/22) of patients treated with ruxolitinib vs 46.7% (7/15) of patients treated with BAT (OR, 2.69; 95% CI, 0.66–10.9; Fig. 2b). Median (range) time to first response for BOR was 4.1 (2.1–15.3) weeks in ruxolitinib and 8.0 (2.1–11.1) weeks in BAT groups.

Two patients in BAT crossed over to ruxolitinib, both achieved PR by crossover week 24. Although interpretation requires caution because of very limited number of patients, response at week 24 was observed in majority of patients independently from organs involved. Patients with skin (45.5% vs 11.1%), mouth (50.0% vs 0%), joints and facia (60.0% vs 33.3%) involvement were the most remarkable responses at week 24 that were observed in comparing ruxolitinib with BAT group (Table 2). Median FFS were 18.6 months in ruxolitinib and 3.7 months in BAT, respectively (HR, 0.34; 95% CI, 0.14–0.85). Kaplan–Meier (KM) estimate of FFS at 6 months was 72.73% in ruxolitinib (95% CI, 49.10–86.71) and 33.33% in BAT (95% CI, 12.15–56.40) (Supplementary Fig. 1). The median OS in ruxolitinib group was 21.9 (95% CI, 3.2, not evaluable [NE]) months and NE (6.7, NE) in the BAT group.

**Table 2:** Organ response rate at week 24 (Full analysis set)

Organ involvement^a^	Ruxolitinib (*n* = 22)	BAT (*n* = 15)
	m/* n* (%)^b^	m/* n* (%)^b^
Skin	5/11 (45.5)	1/9 (11.1)
Eyes	1/12 (8.3)	1/7 (14.3)
Mouth	5/10 (50.0)	0/7
Esophagus	0/1	1/1 (100.0)
Upper GI	0/1	0/2
Lower GI	0	1/2 (50.0)
Liver	2/10 (20.0)	1/9 (11.1)
Lung	1/16 (6.3)	1/8 (12.5)
Joints and fascia	3/5 (60.0)	1/3 (33.3)

Organ response as documented by the investigator, m is the number of patients with organ response = CR or PR and excluding those responders where the organ abnormality is due to non-cGvHD reasons. Overall response counts patients with CR or PR as per investigator

^a^Baseline involvement if respective score at day 1 > 0, or %FEV1 < 75% (lung), ALT, Bili or ALP > ULN (liver), J and F score > 0 based on NIH cGvHD response guidelines [22]

^b^m/n shows number of responders/patients with baseline involvement excluding in m those patients with change/addition of new systemic cGvHD treatment before week 24

*ALP* alkaline phosphatase, *ALT*, alanine aminotransferase, *BAT* best available therapy, *Bili* bilirubin, *cGvHD* chronic graft-versus-host disease, *CR* complete response, *FEV* forced expiratory volume, *GI* gastrointestinal, *J and F score* joints and fascia score, *PR* partial response, *ULN* upper limit of normal

**Fig. 3:**
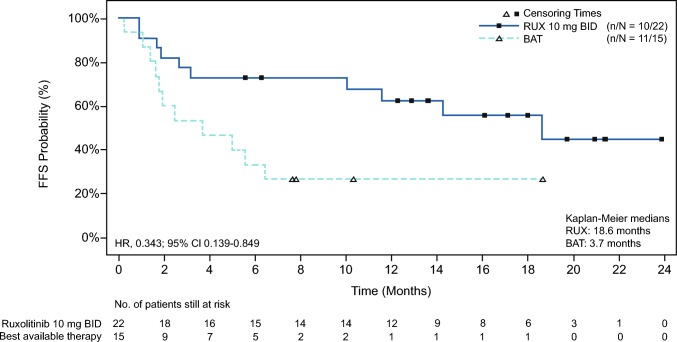
Kaplan–Meier estimate of failure-free survival (FFS) at week 24. *BAT* best available therapy, *BID* twice a day, *FFS* failure-free survival, *RUX* ruxolitinib. FFS is defined as time from the date of randomization to the earliest of recurrence of underlying disease, start of new systemic treatment for cGvHD, or death

**Fig. 4:**
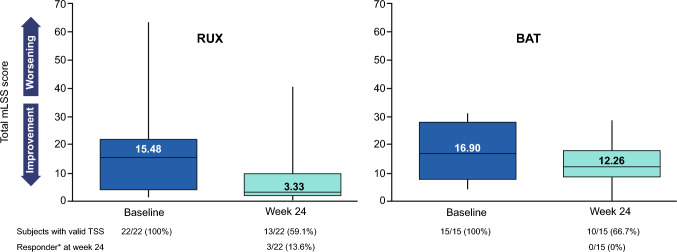
Change from baseline in summary symptom score (mLSS) at week 24. Change from baseline calculated for patients with available data at baseline and the corresponding post baseline timepoint. *Patients with change of or addition of new systemic cGvHD treatment are counted as non-responders irrespective of the TSS value. *BAT* best available therapy, *cGvHD* chronic graft-versus-host disease, *mLSS* modified Lee cGvHD symptom scale, *RUX* ruxolitinib, *TSS* total symptom score

Patient-reported outcomes (PROs) were measured in this study. Modified Lee Symptom Scales, mLSS, is a cGvHD-specific self-administered survey where higher score indicates worse symptoms. At baseline, the median (range) mLSS score in the ruxolitinib (*n* = 22) was 15.48 (1.2–64.0) that was comparable with BAT group (*n* = 15) 16.90 (4.0–31.2). At week 24, median (range) mLSS was improved to 3.33 (0.0–40.7) in ruxolitinib group (*n* = 13), whereas it was 12.26 (0.0–28.9) in BAT group (*n* = 10) (Figs. 3, 4). Three patients in ruxolitinib group (13.6%) achieved clinically meaningful response defined as a ≥ 7-point TSS reduction from baseline at week 24 while none in BAT. Dose of steroid gradually decreased over time in both ruxolitinib and BAT groups, with a slightly faster decrease with ruxolitinib (Supplementary Fig. 2). Other PROs, FACT-BMT and EQ-5D-5L scores, suggested slight improvements of QoL in the patients treated with ruxolitinib, where higher scores suggest improvement of QoL in both scales (Supplementary Table 4). Median DOR was NE (95% CI, 2.2–NE) with ruxolitinib and 5.1 months (95% CI, 1.2–NE) with BAT. KM estimated for DOR at 6 and 12 months were 78.97% (95% CI, 47.91–92.71) and 71.79% (95% CI, 41.11–88.38) with ruxolitinib and 42.86% (95% CI, 9.78–73.44) and NE (95% CI, NE–NE) with BAT groups, respectively.

### Safety

Among the Japanese participants, safety analysis was performed for 37 patients (ruxolitinib = 22, BAT = 15) who received at least one dose of study treatment. The median duration of exposure to therapy up to week 24 was similar between two arms, 25.6 weeks (range 3.0–25.6) in the ruxolitinib group and 25.6 weeks (range 0.6–25.6) in the BAT group, whereas the exposure up to data cut-off was longer in the ruxolitinib group, 44.4 weeks (range, 3.0–112.1) in the ruxolitinib group, and 29.1 weeks (range, 0.6–83.3) in the BAT group. The median dose intensity of ruxolitinib up to week 24 was 19.8 mg/day (range, 8.4–20.5) in Japanese patients which was similar to the overall population (19.6 mg/day, range 4.8–20.5).

Adverse events of any grade up to week 24 are reported in Table 3. In the ruxolitinib group, 13 (59.1%) patients reported at least one grade ≥ 3 adverse event compared with 12 (80.0%) patients in the BAT group. Grade ≥ 3 anemia (5 [22.7%] vs 1 [6.7%]) and pneumonia (5 [22.7%] vs 3 [20.0%]) were the most common adverse events reported in ruxolitinib vs BAT group (Table 3). Serious adverse events up to week 24 occurred in 10 (45.5%) patients treated with ruxolitinib and 8 (53.3%) patients treated with BAT in the study. Up to week 24, adverse events leading to dose adjustment or interruption occurred in 7 (31.8%) and 2 (13.3%) patients and adverse events leading to treatment discontinuation occurred in 9 (40.9%) and 3 (20.0%) in the ruxolitinib and BAT groups, respectively (Supplementary Table 5).

**Table 3:** Most frequent adverse events up to week 24 (occurring in ≥ 10% patients in any group; safety set)

Event,* n* (%)	Ruxolitinib (*n* = 22)		BAT (*n* = 15)	
	Any Grade	Grade ≥ 3	Any Grade	Grade ≥ 3
Number of patients with at least one event^a^	21 (95.5)	13 (59.1)	15 (100.0)	12 (80.0)
Hematologic event				
Anemia	10 (45.5)	5 (22.7)	1 (6.7)	1 (6.7)
Thrombocytopenia	4 (18.2)	4 (18.2)	1 (6.7)	1 (6.7)
Lymphopenia	1 (4.5)	1 (4.5)	2 (13.3)	1 (6.7)
Infections				
Pneumonia	5 (22.7)	5 (22.7)	4 (26.7)	3 (20.0)
Nasopharyngitis	3 (13.6)	0	3 (20.0)	0
Upper respiratory tract infection	3 (13.6)	0	0	0
Cystitis viral	0	0	2 (13.3)	0
Laboratory abnormality				
Alanine aminotransferase increased	4 (18.2)	1 (4.5)	0	0
Aspartate aminotransferase increased	4 (18.2)	1 (4.5)	0	0
Platelet count decreased	4 (18.2)	2 (9.1)	1 (6.7)	1 (6.7)
γ-glutamyl transferase increased	3 (13.6)	2 (9.1)	1 (6.7)	1 (6.7)
Hypokalemia	3 (13.6)	0	2 (13.3)	1 (6.7)
Hyperuricemia	3 (13.6)	0	0	0
Hyperlipidemia	2 (9.1)	1 (4.5)	2 (13.3)	1 (6.7)
Hypogammaglobulinemia	2 (9.1)	0	2 (13.3)	0
White blood cell count decreased	1 (4.5)	0	2 (13.3)	1 (6.7)
Hypoalbuminemia	1 (4.5)	0	2 (13.3)	1 (6.7)
Hypophosphatemia	0	0	2 (13.3)	1 (6.7)
Gastrointestinal event				
Constipation	3 (13.6)	0	0	0
Diarrhea	0	0	3 (20.0)	0
Other				
Pyrexia	3 (13.6)	0	2 (13.3)	0
Hypertension	3 (13.6)	1 (4.5)	1 (6.7)	1 (6.7)
Dehydration	3 (13.6)	0	2 (13.3)	0
Dry eye	3 (13.6)	0	0	0
Headache	2 (9.1)	0	3 (20.0)	0
Epistaxis	1 (4.5)	0	2 (13.3)	0
Acne	0	0	2 (13.3)	0
Non-cardiac chest pain	0	0	2 (13.3)	0

Numbers (*n*) represent counts of patients

^a^A patient with multiple severity grades for an AE is only counted under the maximum grade

*AE* adverse event, *BAT* best available therapy

Up to week 24, infections of any type occurred in 16 (72.7%) of patients in the ruxolitinib group vs 11 (73.3%) in the BAT group, and ≥ grade 3 occurred in 7 (31.8%) vs 2 (13.3%), respectively. Viral infections were the most common (8 [36.4%] vs 8 [53.3%]), followed by bacterial (8 [36.4%] vs 7 [46.7%]), and fungal (5 [22.7%] vs 0; Table 4). At data cut-off, on-treatment death was reported in 5 patients who received ruxolitinib and 2 patients who received BAT, primarily due to complications caused by cGvHD and/or its treatment. Fatal serious adverse events related to study treatment were reported for 4 patients; 3 patients in the ruxolitinib group with pneumonia (*n* = 1), respiratory failure (*n* = 1), pneumonia bacterial (*n* = 1), sepsis (*n* = 1) where pneumonia and respiratory failure had one case reported from a same patient, and 1 patient in the BAT group with pneumonia (*n* = 1). All patients who died had severe cGvHD at baseline. The incidence of cancer relapse and progression was low in both arms (1 patient each).

**Table 4:** Overview of infections up to week 24 (Safety set)

Type of infection,* n* (%)^a^ Maximum severity grade	Ruxolitinib (*n* = 22)	BAT (*n* = 15)
Patients with ≥ 1 event (any type of infection)	16 (72.7)	11 (73.3)
Grade 1	4 (18.2)	4 (26.7)
Grade 2	5 (22.7)	5 (33.3)
Grade 3	7 (31.8)	2 (13.3)
Fungal infections	5 (22.7)	0
Pneumonia (grade 3)	3 (13.6)	0
Brain abscess (grade 3)	1 (4.5)	0
Pneumonia fungal (grade 3)	1 (4.5)	0
Viral infections	8 (36.4)	8 (53.3)
Pneumonia (grade 3)	1 (4.5)	1 (6.7)
Ileus (grade 3)	0	1 (6.7)
Bacterial infections	8 (36.4)	7 (46.7)
Pneumonia (grade 3)	1 (4.5)	1 (6.7)
Pneumonia bacterial (grade 3)	1 (4.5)	0
Sepsis (grade 3)	1 (4.5)	0
Meningitis (grade 3)	1 (4.5)	0

^a^Infections were classified by type (viral, bacterial, fungal, unknown, or other) and severity (grades 1 to 3) at the investigator’s discretion using an infection-specific grading system predictive of mortality that was developed for and validated in allogeneic stem cell transplant recipients [25] based on criteria provided in the protocol. A patient with multiple severity grades for an AE is only counted under the maximum grade

*BAT* best available therapy

## Discussion

Chronic GvHD is one of the leading causes of morbidity, decreased QoL, and mortality in pediatric and adult patients after allogeneic HSCT [26]. Immunosuppressants are suggested as a treatment option although with limited evidence from well-designed randomized studies. A standard second-line treatment is not well established for cGvHD [27]. Hence, there is a high unmet medical need for new treatment options that can be effective, well tolerated in both adult and pediatric cGvHD patients [26]. REACH3 is a phase 3 randomized study that showed the superiority of ruxolitinib over common second-line therapeutic options, including ibrutinib and ECP, for treatment of SR/D cGvHD [18]. Japanese patients in this study included more severe cGvHD patients and patients with lung involvement at baseline than overall population. Ibrutinib and MMF were common BATs chosen by Japanese investigators whereas ECP was major in whole study. The subgroup analysis by Japanese population showed favorable results of ruxolitinib over BAT and were consistent with the overall population [19]. Ruxolitinib demonstrated an increased ORR (50% vs 20%), longer FFS (18.6 vs 3.7 months), better mLSS response (13.6% vs 0%) and a higher BOR up to week 24 (68.2% vs 46.7%) than BAT, respectively. These results are in line with the global REACH3 results for overall population [19].

As one of the treatment goals for cGvHD is intended to produce a sustainable benefit by reducing symptom burdens without producing disproportionate harms related to the treatment itself, PROs are important components to assess the efficacy of cGvHD treatment and are recommended by the NIH consensus criteria for clinical trials in cGvHD [21]. Reduction of the median symptom score was observed among ruxolitinib-treated patients whereas the change was mild in the BAT. Three patients in the ruxolitinib were symptom responders by mLSS at week 24. All mLSS responders also achieved physician-assessed cGvHD responses, CR (*n* = 1) and PR (*n* = 2), at week 24. Although with a limited number of patients, these results suggest that symptom assessment by mLSS can be used in Japanese cGvHD patients and it was associated with clinical improvements assessed by physicians. The limitation of mLSS in this study was to assess patients who showed mild symptoms at baseline. Of note, 7/22 patients in the ruxolitinib and 2/15 patients in the BAT had summary symptom score below 7 points at baseline. Thus, they were symptom non-responders at week 24. The median baseline symptom score in Japanese was slightly lower than overall population although there were more severe cGvHD patients. It can reflect lower symptom responder rate in both arms in Japanese patients than the overall population.

The daily dose of ruxolitinib was set as 20 mg (10 mg BID) in this study. The median dose intensity up to week 24 in Japanese patients was 19.8 mg per day which was similar to the overall population [19]. The duration of exposure up to data cut-off was also similar to the overall population and it was longer in the ruxolitinib group than the BAT group, which was reflected in the KM estimate of FFS in terms of other events with higher rate of treatment change in BAT group and higher rate of non-relapse mortality in ruxolitinib group. Cytopenias, such as neutropenia, anemia, and thrombocytopenia, as well as infections were major adverse events leading to ruxolitinib dose adjustments. Of note, ruxolitinib dose adjustments were mandatory for patients who had treatment-related grade 3/4 neutropenia and grade 4 thrombocytopenia per study protocol. Most cytopenia events were manageable with ruxolitinib dose adjustments or interruption, and there was no cytopenia event leading to ruxolitinib discontinuation reported in Japanese patients. These data suggest that ruxolitinib treatments were well tolerated in Japanese patients with the current initial dose and dose adjustments.

Infections were the most common adverse events reported in overall REACH3 study population in both ruxolitinib and BAT groups. In Japanese patients, grade 3 infection rates were also commonly reported in both groups. Bacterial and viral infections were common types of infections reported similarly in both groups. Incidence of fungal infections was higher with ruxolitinib compared to BAT and similar trend was observed in the overall population. Given such risks of infections, prophylaxis against infections is recommended in ruxolitinib-treated cGvHD patients. A total of 4 deaths in Japanese patients were reported as treatment-related, 3 with ruxolitinib and 1 BAT therapy, which could be reflective of more severe patient population in Japanese analysis.

The PK parameters and trough concentrations were obtained in Japanese patients and the results were almost within the range in non-Japanese patients, suggesting PK ethnic insensitivity consistent with the results in healthy volunteers and patients in the other indications (myelofibrosis and polycythemia vera).

In this study, three Japanese adolescent patients from 12 to 18 years old were included. Two of them were treated with ruxolitinib, one in ruxolitinib group and one in crossover. Two adolescent patients achieved PR after receiving ruxolitinib without major adverse event. Grade 3 or higher adverse event reported from ruxolitinib-treated adolescent patients was grade 3 herpes zoster (*n* = 1). Both patients continued ruxolitinib treatment at the time of data cut-off. PK differences between adolescent and adult patients were not indicated (data not presented).

The major limitation of this analysis was the small sample size of Japanese population; the subgroup analysis was not designed to perform any hypothesis testing or to derive any conclusion.

In summary, the current subgroup analysis demonstrates that patients with moderate or severe cGvHD who had an inadequate response to steroids, showed better outcomes (ORR, BOR, longer FFS, and greater symptom improvement) and tolerability with ruxolitinib treatment over BAT in Japanese population. These results are in line with the overall population of REACH3 study.

## Data sharing

Novartis is committed to sharing with qualified external researchers, access to patient-level data and supporting clinical documents from eligible studies. These requests are reviewed and approved by an independent review panel on the basis of scientific merit. All data provided are anonymized to respect the privacy of patients who have participated in the trial in line with applicable laws and regulations. The availability of this trial data is according to the criteria and process described on www.clinicalstudydatarequest.com.

## Supplementary Information

Below is the link to the electronic supplementary material.

Supplementary file1 (DOCX 2767 kb)
