# Supplementary material for: Ruxolitinib for steroid-refractory chronic graft-versus-host disease: Japanese subgroup analysis of REACH3 study
Source: Int J Hematol. 2024 Oct 3;120(6):705–16. doi: 10.1007/s12185-024-03850-9 (PMC11588829; doi:10.1007/s12185-024-03850-9)
Supplement: Supplementary file 1 — Supplementary file1 (DOCX 2767 kb) [file 12185_2024_3850_MOESM1_ESM.docx]

Supplementary Table 1. Prior medications and organ baseline scores at baseline (Full analysis set)

| **Characteristic** | **Ruxolitinib**  **(n=22)** | **BAT (n=15)** | **Total (N=37)** |
| --- | --- | --- | --- |
| **Prior systemic therapy for cGvHD or**  **glucocorticoid-refractory/dependent**  **cGvHD, n (%)^a^** |  |  |  |
| Glucocorticoid only | 6 (27.3) | 4 (26.7) | 10 (27.0) |
| Glucocorticoid + CNI | 14 (63.6) | 9 (60.0) | 23 (62.2) |
| Glucocorticoid + CNI + other systemic therapy | 0 | 1 (6.7) | 1 (2.7) |
| Glucocorticoid + other systemic therapy | 2 (9.1) | 1 (6.7) | 3 (8.1) |
| **Organ involvement, n (%)^b^** |  |  |  |
| Lung |  |  |  |
| Any | 15 (68.2) | 10 (66.7) | 25 (67.6) |
| With score =2 | 9 (40.9) | 4 (26.7) | 13 (35.1) |
| With score =3 | 4 (18.2) | 2 (13.3) | 6 (16.2) |
| Skin |  |  |  |
| Any | 11 (50.0) | 9 (60.0) | 20 (54.1) |
| With score ≥2 | 10 (45.5) | 8 (53.3) | 18 (48.6) |
| Eyes |  |  |  |
| Any | 12 (54.5) | 7 (46.7) | 19 (51.4) |
| With score ≥2 | 6 (27.3) | 4 (26.7) | 10 (27.0) |
| Mouth |  |  |  |
| Any | 9 (40.9) | 7 (46.7) | 16 (43.2) |
| With score ≥2 | 1 (4.5) | 4 (26.7) | 5 (13.5) |
| Joints and fascia |  |  |  |
| Any | 5 (22.7) | 2 (13.3) | 7 (18.9) |
| With score ≥2 | 3 (13.6) | 2 (13.3) | 5 (13.5) |
| Liver |  |  |  |
| Any | 4 (18.2) | 2 (13.3) | 6 (16.2) |
| With score =2 | 2 (9.1) | 0 | 2 (5.4) |
| With score =3 | 1 (4.5) | 1 (6.7) | 2 (5.4) |
| Gastrointestinal |  |  |  |
| Any | 1 (4.5) | 5 (33.3) | 6 (16.2) |
| With score ≥2 | 1 (4.5) | 3 (20.0) | 4 (10.8) |
| Genital tract |  |  |  |
| Any | 0 | 1 (6.7) | 1 (2.7) |
| ^a^Prior treatment for cGvHD as documented in the prior medication data; topical or local treatments not  counted.  ^b^Organ involvement at baseline was based on NIH consensus staging criteria at screening. A score of  one or greater is counted as organ involvement. Patients with missing assessments of single organs are  counted as having no organ involvement for the organ assessed.  BAT, best available therapy; cGvHD, chronic graft-versus-host disease; CNI, calcineurin inhibitor. | | | |

Supplementary Table 2: Initial BAT treatment (Safety set)

| **Initial BAT treatment (n%)** | **BAT**  **(n=15)** |
| --- | --- |
| Ibrutinib | 6 (40.0) |
| Mycophenolate mofetil | 6 (40.0) |
| Imatinib | 2 (13.3) |
| Rituximab | 1 (6.7) |

BAT, best available therapy.

Supplementary Table 3: Underlying disease history by treatment (Full analysis set)

| **Disease history** | **Ruxolitinib**  **(n=22)** | **BAT**  **(n=15)** | **All patients (N=37)** |
| --- | --- | --- | --- |
| **Primary diagnosis classification (n%)** |  |  |  |
| Malignant-leukemia/Myelodysplastic syndrome | 21 (95.5) | 12 (80.0) | 33 (89.2) |
| Malignant-lymphoproliferative disorder | 1 (4.5) | 2 (13.3) | 3 (8.1) |
| Non-malignant-severe aplastic anemia | 0 | 1 (6.7) | 1 (2.7) |
| **Diagnosis of underlying malignant disease (n%)** |  |  |  |
| Acute lymphoblastic leukemia | 2 (9.1) | 1 (6.7) | 3 (8.1) |
| Acute myelogenous leukemia | 14 (63.6) | 6 (40.0) | 20 (54.1) |
| Chronic myelogenous leukemia | 0 | 2 (13.3) | 2 (5.4) |
| Myelodysplastic disorder | 5 (22.7) | 3 (20.0) | 8 (21.6) |
| Non-Hodgkin lymphoma | 1 (4.5) | 0 | 1 (2.7) |
| Other | 0 | 2 (13.3) | 2 (5.4) |
| **Disease history** | **Ruxolitinib**  **(n=22)** | **BAT**  **(n=15)** | **All patients (N=37)** |
| **Primary diagnosis classification (n%)** |  |  |  |
| Malignant-leukemia/Myelodysplastic syndrome | 21 (95.5) | 12 (80.0) | 33 (89.2) |
| Malignant-lymphoproliferative disorder | 1 (4.5) | 2 (13.3) | 3 (8.1) |
| Non-malignant-severe aplastic anemia | 0 | 1 (6.7) | 1 (2.7) |
| **Diagnosis of underlying malignant disease (n%)** |  |  |  |
| Acute lymphoblastic leukemia | 2 (9.1) | 1 (6.7) | 3 (8.1) |
| Acute myelogenous leukemia | 14 (63.6) | 6 (40.0) | 20 (54.1) |
| Chronic myelogenous leukemia | 0 | 2 (13.3) | 2 (5.4) |
| Myelodysplastic disorder | 5 (22.7) | 3 (20.0) | 8 (21.6) |
| Non-Hodgkin lymphoma | 1 (4.5) | 0 | 1 (2.7) |
| Other | 0 | 2 (13.3) | 2 (5.4) |

BAT, best available therapy.

Supplementary Table 4: Treatment group and PRO measures

| **Variable** | **Ruxolitinib**  **(n=22)** | **BAT**  **(n=15)** |
| --- | --- | --- |
| **FACT-BMT score, mean (SD)** | | |
| Baseline | 83.39 (23.806) | 90.50 (16.212) |
| At week 24 (ruxolitinib, n=14; BAT, n=10) | 96.73 (23.030) | 89.20 (19.399) |
| Change from baseline at Week 24 | 1.06 (14.212) | –2.48 (17.005) |
| **EQ-5D-5L score, mean (SD)** | | |
| Baseline | 0.61 (0.428) | 0.69 (0.321) |
| At week 24 (ruxolitinib, n=14; BAT, n=10) | 0.84 (0.251) | 0.70 (0.296) |
| Change from baseline at Week 24 | 0.01 (0.143) | –0.05 (0.286) |

BAT, best available therapy; EQ-5D-5L, EuroQoL 5-dimension 5-level; FACT-BMT; functional assessment of cancer therapy – bone marrow transplant; PRO, patient-reported outcomes; SD, standard deviation.

Supplementary Table 5: AEs up to week 24 leading to treatment discontinuation

|  | **Ruxolitinib**  **(n=22)** | | **BAT**  **(n=15)** | |
| --- | --- | --- | --- | --- |
| **Preferred term, n (%)^a^** | **Any Grade** | **Grade ≥3** | **Any Grade** | **Grade ≥3** |
| Hematologic event |  |  |  |  |
| White blood cell count decreased | 0 | 0 | 1 (6.7) | 0 |
| Gastrointestinal disorders |  |  |  |  |
| Ileus | 1 (4.5) | 1 (4.5) | 0 | 0 |
| General disorders and administration site conditions |  |  |  |  |
| Multiple organ dysfunction syndrome | 0 | 0 | 1 (6.7) | 1 (6.7) |
| Infections and infestations |  |  |  |  |
| Pneumonia | 4 (18.2) | 4 (18.2) | 1 (6.7) | 1 (6.7) |
| Brain abscess | 1 (4.5) | 1 (4.5) | 0 | 0 |
| Pneumonia bacterial | 1 (4.5) | 1 (4.5) | 0 | 0 |
| Sepsis | 1 (4.5) | 1 (4.5) | 0 | 0 |
| Neoplasms benign, malignant and unspecified |  |  |  |  |
| Post transplant lymphoproliferative disorder | 1 (4.5) | 1 (4.5) | 0 | 0 |
| Respiratory/thoracic and mediastinal disorders |  |  |  |  |
| Pneumothorax | 1 (4.5) | 1 (4.5) | 0 | 0 |
| Respiratory failure | 1 (4.5) | 1 (4.5) | 0 | 0 |
| Numbers (n) represent counts of patients.  ^a^A patient with multiple severity grades for an AE is only counted under the maximum grade.  AE, adverse event; BAT, best available therapy. | | | | |

Supplementary Figure 1: Failure-free survival (FFS) by treatment

**
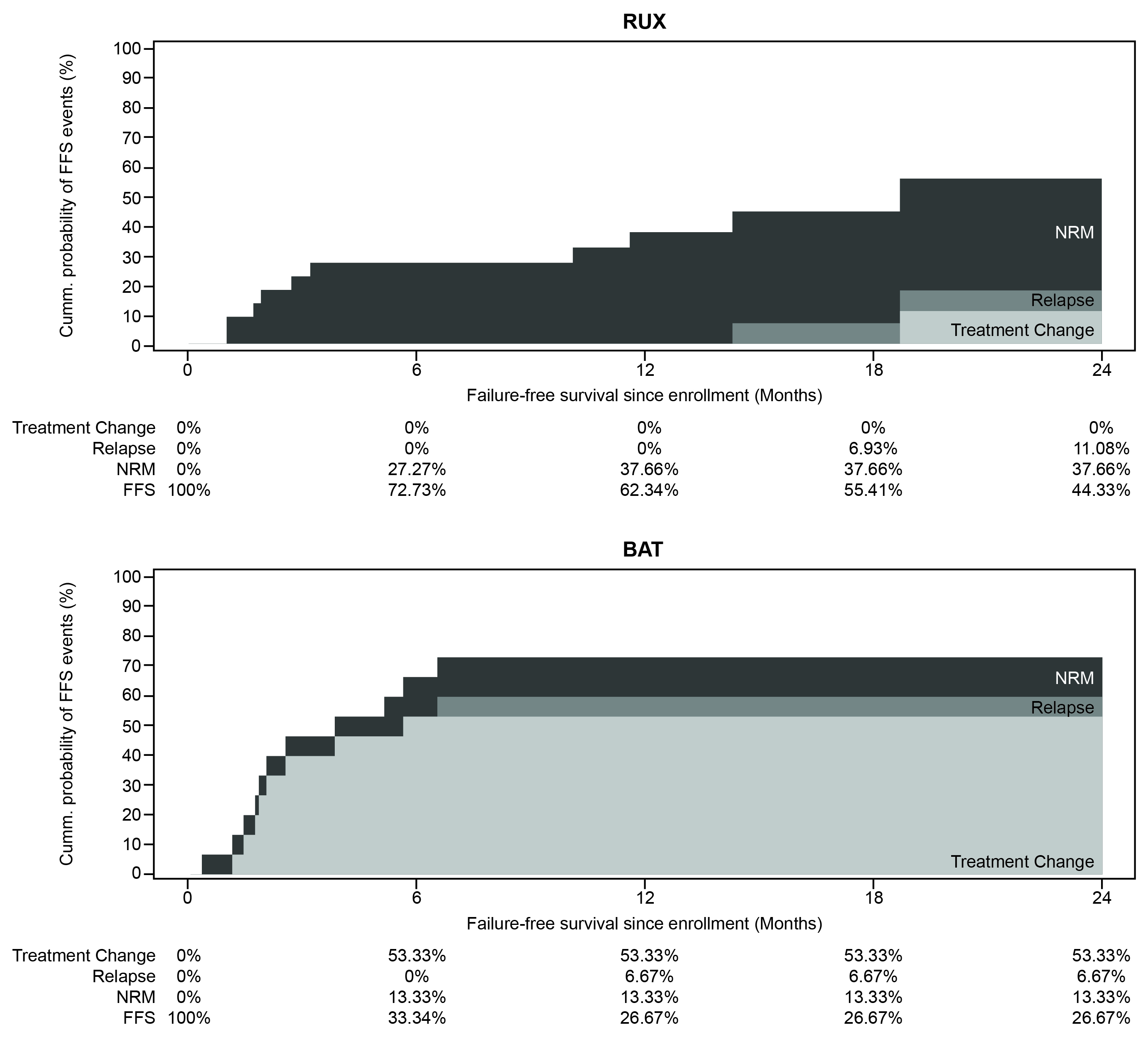
**

BAT, best available therapy; FFS, failure free survival; NRM, non-relapse mortality; FFS probabilities were obtained by 100% - sum of the probabilities for Treatment change, Relapse and NRM.

Supplementary Figure 2: Average biweekly steroid dosing reduction


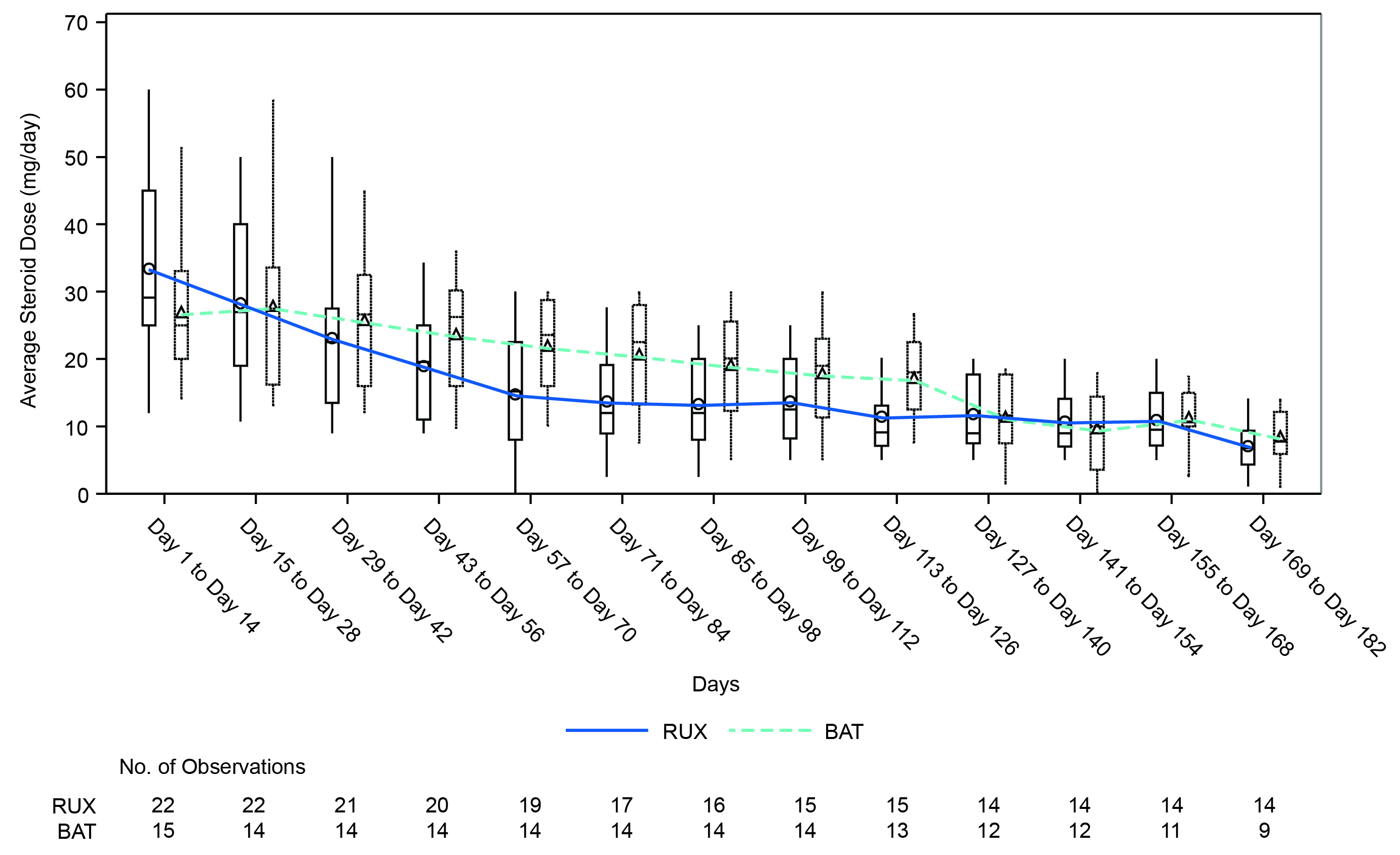


Patients who are completely tapered off steroids and are ongoing were counted as having steroid dose=0 until the end of the main treatment period or the re-start of treatment with systemic steroids. Plot showing boxes (25th-75th percentiles) with median as horizontal line. The dots in the boxes and joint lines represent the mean values. Whiskers (vertical lines) extend to the 10th-90th percentiles. Values outside this range are not displayed. Dose of methylprednisolone was converted to prednisone equivalent.

BAT, best available therapy; RUX, ruxolitinib.
